# The Health Experiences of Young Internal Migrants in Ghana—Identifying Priorities for Sustainable Health Promotion

**DOI:** 10.3390/ijerph192215229

**Published:** 2022-11-18

**Authors:** Grace Spencer, Ernestina Dankyi, Jill Thompson, Faye Acton, Stephen Owusu Kwankye

**Affiliations:** 1 Faculty of Health, Education Medicine and Social Care, Anglia Ruskin University, Cambridge CB1 1PT, UK; 2 Centre for Social Policy Studies, University of Ghana, Legon, Accra LG 1181, Ghana; 3 Health Sciences School, University of Sheffield, Sheffield S10 2HQ, UK; 4 Regional Institute for Population Studies, University of Ghana, Legon LG 1181, Ghana

**Keywords:** health promotion, migration, young people, migrants, sustainable development goals (SDGs), social determinants of health, vulnerability, health inequalities

## Abstract

The Sustainable Development Goals underscore the importance of migration to the achievement of health, and global migration presents both opportunities and challenges for the development of health promotion. Despite such recognition, very little work has focused on health promotion with young migrants, including how migration shapes opportunities for positive health. This paper reports findings from a qualitative study that sought to advance knowledge of the health experiences of young internal migrants in Ghana (*n* = 14) and considers ways to harness these perspectives in the development of sustainable health promotion solutions. Methods included community consultations, participatory workshops and interviews with young migrants aged 14–21 years. Findings highlighted how the social determinants of health affected young migrants’ opportunities to support their health. Our analysis highlights how a lack of access to adequate food, shelter and health services often resulted in the adoption of alternative health practices, including the use of herbal remedies. Supporting positive livelihoods as part of tackling the social determinants of health is crucial to mitigate the impacts of poverty and inequalities on young migrants’ health practices and outcomes. We conclude by considering how to advance relevant health promotion with young migrants living in contexts of vulnerability.

## 1. Introduction

Significant changes to global patterns of migration present opportunities and challenges for the development of sustainable livelihoods and positive health. The United Nations (UN) Sustainable Development Goals (SDGs) underscore the importance of migration to the achievement of health and social equity (e.g., Goals 1–6, 8, 10 and 16 [1]). Despite such recognition, very little work has focused on the vulnerabilities and inequities experienced by children and young people (below the age of 24 years) who migrate (both internally and across national borders)—and crucially, from their own perspectives; [2,3,4] including how migration decisions and journeys shape opportunities for positive health and social inclusion. Even less work has focused on the opportunities for health promotion with migrant groups or indeed explored the health-enhancing or hindering practices that young migrants engage with in their everyday lives (some exceptions include [5,6]).

Migration is increasingly recognised as a key social determinant of health [7]—often because of the complexities of migration and resettlement processes that have been found to affect both physical and mental health of migrant groups [8]. Yet, little work has sought to examine the relationship between mobility, health and poverty—particularly for young people [9]. Evidence to date suggests that migrant groups are often exposed to a range of risks and vulnerabilities including poor living and working conditions, lack of access to adequate shelter, food and health care, coupled with exposure to exploitation, discrimination and abuse [6]. Inevitably, such exposures have been found to result in poorer health status and unmet health needs [10], with migrants at greater risk of communicable diseases and mental health issues.

Despite evidence demonstrating increased health risks for migrant population groups, other research suggests a counter-perspective highlighting the positive health of migrants [11]. In part, this ‘migration paradox’ has been explained by the fact that those with poorer health are less likely to move or have less access to the necessary resources to assist migration, such as economic and social capital. However, this body of evidence also suggests that the relative positive health status of migrants declines following migration —due to increased exposure to health risks, including a lack of access to health care and experiences of discrimination as previously described [12,13,14,15]. 

Accurate data about the health status of young migrants are often fragmented and difficult to decipher, with little research taking an explicit focus on the health of children and young people who migrate, particularly in Western Africa [2,3] (we discuss some exceptions shortly). We have documented elsewhere some of the challenges linked to establishing the health of migrant children and young people [2,3], often because young people’s health and migration status are conflated with that of adults (most notably parents), [2,4,16], but also because of the difficulties tied to establishing migrant children and young people’s exact ages [2,4]. Evidence that does exist highlights how young migrants (both internal and international migrants) are especially vulnerable to poorer health outcomes including declining mental health, as well as increased exposure to health risk factors (e.g., poverty, malnutrition, discrimination, violence). Such evidence prompts urgent calls for health promotion to address the health of young migrants—yet, little attention has been given to the best ways to promote the health of migrant groups or indeed, has considered the role of health promotion in securing positive health for migrants [17,18]. There has been some recent interest in this area [19], although more attention is required in specific African contexts and with young migrants.

Some recent evidence from Europe suggests an absence of knowledge about how best to engage migrants in health promotion, with calls for urgent policy responses such as those proposed by the World Health Organisation [WHO] [20] to enhance culturally relevant and effective approaches to health promotion [18]. Yet, little work has focused specifically on health promotion with young migrants [2]. Health promotion takes many forms ranging from education and behaviour change programmes that typically target individual motivations and practices [21] to community responses and political advocacy work and empowerment models that aim to address socio-political and economic influences on health [22]. Indeed, health promotion has at its heart the development of policy solutions that enable individuals and communities to gain control over the influences on their lives in order to improve their health, which is fundamental to the achievement of health and social equity [22,23]. More recently, the Shanghai Global Conference on Health Promotion [24] has underscored the role of health promotion to the achievement of the SDGs (especially Goal 3)—placing particular emphasis on inter-sectoral collaboration and partnerships for the achievement of health and social equity [25]. Despite such emphasis, health promotion with migrant groups and especially migrant youth remains underdeveloped.

To address these gaps, in this paper we report findings from a qualitative study that sought to ascertain the health-related perspectives and experiences of young migrants aged 14–21 years in Ghana. In keeping with empowerment-based approaches to health promotion [22,26], we aimed to identify how migrants understand health, and their own suggestions for the best ways to support their health, as a necessary first step towards the development of culturally relevant health promotion approaches, which match the everyday lives and experiences of young migrants. The project’s main aim was twofold:

- To advance knowledge of the health-related experiences and perspectives of young migrants in Ghana;
- Harness these perspectives to inform the development of culturally informed sustainable health promotion solutions.

We used the internationally agreed definition of migrant as; ‘a person who moves away from his or her place of usual residence, whether within a country or across an international border, temporarily or permanently, and for a variety of reasons’ [27]. Such movement can be voluntary or involuntary.

Before detailing our study materials and methods, we provide a short overview of the context in which the research took place—namely Ghana and review the (limited) extant evidence to date on the health of young migrants in Ghana.

## 2. Migration and Health in Ghana

In Ghana, over a third (35.3%) of the population are under the age of 25 years [28], with evidence suggesting that many young people live in contexts of poverty and experience poor health outcomes (especially in relation to mental health, sexual health, and communicable diseases) [29]. With a relatively stable political and economic regime within a region of political instability, Ghana is increasingly becoming an attractive destination for young migrants from Western Africa to the extent that there are currently large communities of Nigerians and other West African nationals in some of the peri-urban localities in the capital city, Accra. In addition, internal migration from northern to southern Ghana has resulted in migration from poorer rural communities to urban centres, with young people often moving to cities in search of work, educational opportunities and a better standard of living [30]. Increases in the numbers of girls and young women migrating to cities have been particularly pronounced, with many girls taking-up work as head-porters (known as Kayayei). Many of these girls are poorly educated and are often exposed to multiple intersecting disadvantages that affect their health [6,31].

Poor work and housing conditions place young migrants (both internal and international) at greater risk of contracting communicable diseases including malaria and sexually transmitted infections [6,30]. Access to health care services is also limited with evidence suggesting that few migrants in Ghana have access to appropriate health insurance and are unable to register for the National Health Insurance Scheme (NHIS) [32,33]. Inevitably, this lack of access to formal health care has been found to trigger alternative health-seeking practices such as the use of home and herbal remedies, in addition to seeking out material and financial support from social networks—especially friends [5,9,34]. For example, social networks have been found to offer a source of food for internal young migrants unable to afford something to eat [5,9,34]. These forms of social capital have been investigated for their positive impacts on young migrants’ resilience and may offer a useful starting point for developing asset-based forms of health promotion [35]. It is to be noted that because cross-border young migrants are largely irregular by nature, they tend to have little or no education, and often with no formal employable skills, which is similar to the backgrounds of many young internal migrants. Because of this, young international and internal migrants, have been found to share similar vulnerabilities and face similar socio-economic challenges, with likely implications for their health.

## 3. Materials and Methods

The project was undertaken between September 2019 and June 2021 and during the time of the COVID-19 pandemic (in line with in-country guidance at that time). Significant disruptions and delays were thus encountered and some adaptations to our methods and processes were required. In keeping with a participatory community-action based approach [22], our original intention was to develop a participatory study in collaboration with local community groups and young people, utilising a range of creative methods including workshops and walking interviews with photo-elicitation. However, social distancing measures and restrictions on ‘free’ movement’ limited many of our participatory intentions. In-country guidelines did, however, allow us to host two workshops with a range of stakeholders including community partners and young migrants, and a series of follow-up interviews with young migrants aged 14–21 years. Here, we report findings from the interviews with young people.

### 3.1. Participants and Recruitment

Study participants included young migrants aged between 14 and 21 years (*n* = 14). Eight young men and six young women took part. All young participants had migrated to the capital city Accra from other regions of Ghana, primarily Northern Ghana. Participants were recruited via our established contacts with social workers from local NGOs supporting young migrants and other vulnerable youth (see Section 4 below). These social workers from NGOs, however, had no influence on the interviews and subsequent responses from study participants.

### 3.2. Data Collection

The project consisted of three main activities: (1) a policy and evidence review, (2) two participatory workshops, and (3) semi-structured interviews with young migrants. The first workshop took place in November 2020 in Ghana with key stakeholders (academics/researchers, policymakers, NGOs, community groups and young people). The aim of the workshop was to:

- Foster dialogue and share knowledge on migration, health, and young people.
- To identify community priorities and solutions that aim to enhance the health of young migrants in Ghana.
- To develop an understanding of the challenges and opportunities in supporting the health of young migrants and to identify priorities for health promotion.

Workshop participants included young migrants, academic researchers, participating NGOs and local Child Protection Leads. The workshop involved a series of presentations followed by group discussions to explore the challenges and opportunities for supporting young migrants and identify priorities for health promotion with young migrants. During the workshop, young people were asked to create a ‘life map’—a qualitative narrative visual method often used in the health and social sciences to depict an individual’s life story over a specified period [36]. As part of this method, the researcher asked participants open-ended questions about their life history while each participant used pens and paper to draw or ‘map’ significant moments in their life in relation to the topic being explored [37]. Life maps were chosen as an approach to help participants reflect on their migration journeys (pre-migration, during and post migration). The content is directed by young people to highlight issues that they feel are particularly pertinent. This illustrative approach was deemed especially appropriate as English was not the first language for many of the participants. Participants were asked to draw their life as a young migrant in Accra. They were asked to include key events and important memories in chronological order. They were also asked to include any health challenges and dangers (including risks to their health) they had experienced. The life maps were then used as prompts during subsequent interviews.

A second follow-up workshop was held in June 2021. The main aim of the second workshop was to use the evidence gathered from workshop one and interviews with young people to identify priorities and strategies for community-based health promotion with young migrants. The same participants that attended workshop one were invited back for the second workshop. This second workshop included presentations that summarised key discussions and ideas from workshop one and findings from the interviews with young people. Group discussions were held to discuss the findings from the interviews and develop ideas for new approaches to health promotion with young migrants. Key discussion questions included:

- What forms of health promotion are needed to support young migrants’ health?
- How can we achieve better health for young migrants?
- What are the top priorities for health promotion and how can we address these?

### 3.3. Interviews

As described, 14 young internal migrants took part in face-to-face interviews (conducted by [ED] and local research assistants) at different locations in Accra, at the convenience of each participant. Interviews with young migrants were conducted in the participants’ native language of Twi, audio recorded and then later transcribed by research assistants at the University of Ghana. The interviews lasted approximately half an hour and covered topics such as why they migrated, their thoughts about their health and what they do to keep healthy, what makes it difficult for them to stay healthy and what help or support they feel they need with their health.

### 3.4. Data Analysis

Interview data were analysed thematically based on the approach by Braun and Clarke [38]. Verbatim transcripts were (re)read and descriptive codes attached to text to capture meaning by three researchers (FA, FS, JT). The codes were discussed by the whole team and then refined to develop categories that helped to organise and group key aspects of the data. These categories were scrutinised to identify and develop core thematic areas from the data. Themes were discussed by the full team to confirm consistency in analytical categories and interpretations, in addition to identifying alternative perspectives on the data. Data were also analysed by gender to consider differences and/or similarities across the perspectives and experiences of young men and young women. Our analysis that follows reflects some important gendered aspects of the data and the health-related and migration experiences of our participants.

## 4. Ethical Considerations

Research ethics approval was granted from a UK University Faculty Research Ethics Panel and the University of Ghana’s Ethical and Protocol Review Committee. A few ethics amendments were made in the course of the study because of the impacts of the COVID-19 pandemic. For example, restrictions on international travel meant hosting part of the workshop online and ensuring all data collection followed in-country COVID-19 guidance (e.g., social distancing meant that we reviewed the number of participants attending the workshops and we were unable to conduct walking interviews and photo-elicitation work as originally planned).

All participants were sent a study information sheet and consent form prior to the workshop and interviews. Potential participants were encouraged to contact the research team for any queries about the study and their participation in the workshop and/or interviews. The research team were available to talk through any queries participants may have had. Participants under the age of 18 years were asked to sign an informed assent form (in line with ethics guidance from the University of Ghana), with written consent ascertained from the respective Directors/Heads of the participating NGOs in loco parentis and when parents were absent. This approach was required by the ethics committee to ensure legal consent was ascertained; in addition to ensuring that participants were fully informed of the research and had access to local support should they need further help.

Talking about health and migration may constitute a sensitive topic area with the potential to cause some discomfort and unease, especially for participating young migrants. To help guard against this, participants were advised that their participation was entirely voluntary and that they were free to withdraw from the workshop or interview at any point without providing a reason. In the context of an interview, should a participant appear uncomfortable or upset, the interview was to be paused and possibly terminated. The participant would have been advised to seek support from a local community organisation or a psychologist the research team had arranged to assist as required/appropriate.

All data were stored in line with data protection legislation and secured in password-protected files. Interview recordings were deleted from the digital recorder and all transcripts were fully anonymised (e.g., removal of any names and all contextual identifiers). A small honorarium was offered to all participants to cover local travel costs and opportunistic costs (e.g., loss of income) to be able to take part in the study.

## 5. Results

Our interviews with young migrants revealed important insights into their health and wellbeing, which may signal new opportunities to develop health promotion directly tailored to their needs and experiences. In particular, participants described the impacts of living in poverty on their health—often reporting significant hardships and vulnerabilities. Lack of access to food, shelter and a safe space to sleep were described as negatively influencing health. Symptoms such as stomach aches, headaches and skin rashes were commonly reported—despite participants’ frequent claims that they had ‘no health issues’. On closer examination, the latter seemed to mean that participants had no access to (funding for) formal health care to seek out a diagnosis and treatment—rather than the actual absence of illness. The use of alternative treatments, such as herbal remedies, were common and discussed positively as one way to support their health. Friends also played a key role in supporting their health through providing financial support for food and home remedies. Other health-enhancing practices included regular exercise and eating healthy foods such as fruits. However, opportunities for these young migrants to promote their health in such ways were seriously constrained by the ongoing effects of living in poverty—highlighting the critical role the social determinants had on these young people’s health as our following analysis reveals.

### 5.1. Young Migrants’ Health Experiences

As part of the interview, participants were asked to describe their experiences of migration including their motivations for moving to the city and how their migration shaped their health. Perhaps unsurprisingly, all participants shared their economic reasonings for migration and their aspirations to come to a larger city and find meaningful employment to enhance their lives. Such hopes were often set on a background of extreme hardship in rural communities and/or difficulties with families. Indeed, family relationships often appeared fractured and the absence of parents and/or experiences of maltreatment by family members triggered these young people’s decision to migrate independently.

“I was staying with my mother, but I don’t know my father. I am the only child of my mother; it was when she died that I moved. My family members claimed my mother was a witch and through that she died. After her death […] I ran away from them”

(Young women #1, aged 15).

“The way my mother was treating me I feel she hates me; it was only my grandmother who cared for me, and my grandmother is dead now so I can’t stay with my mother because she can just get angry”

(Young man #4, aged 17).

Because of these difficult family relationships, overwhelmingly, migration was viewed positively—offering new opportunities for a better life and away from the hardships they had experienced. However, despite aspirations for a better future, many participants also described how life in the city was hard and they continued to suffer. In particular, a lack of access to adequate shelter or housing, sanitation and employment often meant that participants were unable to afford basic necessities to support their health and were exposed to a range of health risks, including malaria and lack of adequate nutrition. Participants described the vulnerabilities they experienced because of poor economic circumstances, including exposure to theft and other crimes—often reporting how life in the city can be ‘hard’. Similarly, participants spoke of the health challenges they faced due to their lack of proper shelter for accommodation, coupled with difficulty in finding jobs to raise reasonable incomes to support them to rent suitable housing. Poor housing and shelter exposed these young people to mosquitoes and frequent episodes of malaria.

Some of their accounts were also quite conflicting. As a young man of 14 years recounted, aside malaria, he did not have any sickness such as headache, but interestingly described how he often felt dizzy. He associated this with being hungry and not sickness because he would always ask his friends for money to buy food whenever he felt dizzy. This suggests that their conception of what constitutes sickness relates largely to getting malaria and that dizziness may not be considered a symptom of sickness or ill-health.

“Things that make it difficult for me to stay healthy are that I don’t have a place to sleep, and I don’t have any work. I came to Accra with the aim to work and make money […]. (W)hen we sleep outside the mosquitoes have been biting us and when you don’t take care other people will steal your mattress and blanket. We don’t even sleep […]. Living in Accra is difficult, getting GHc1.00 to pay for bathing gets difficult, and also don’t get food to eat sometimes”

(Young woman #3, aged 18).

The above highlights some of the negative, and perhaps unanticipated, influences on participants’ health that came to the fore post-migration. Despite these concerns, participants concurrently stated that they had no health complaints—all the while sharing examples of times when they had fallen sick. When probed further about their health, a range of vague symptoms was reported, suggesting that many of these young people were experiencing some health issues. Descriptions of gastro-intestinal symptoms, skin conditions and headaches were common—as well as genital-urinary symptoms that suggested the presence of a sexually transmitted infection.

“I have not experienced any sickness like malaria or diarrhoea. The only sickness that I usually get is headache. The headache usually comes in the afternoon when I am working in the sun”

(Young man #2, aged 19).

Symptoms of this kind were often attributed to a lack of food, which caused dizziness and headaches. Similarly, eating contaminated food available from street sellers was identified as a cause for stomach complaints and gastro-intestinal symptoms. Yet, these symptoms were often ‘explained away’ as participants continued to suggest that they had no health issues. In part, the tendency for participants to downplay any symptoms might be linked to their concerns about stigma, shame and embarrassment—particularly when health complaints were linked to ‘risky’ health behaviours. For example, some participants underscored how peers can influence health and health practices (see Section 5.3) but also how their socio-economic circumstances dictated opportunities to be healthy and/or access health care treatment for symptoms.

“I have some sickness that if I tell you, you will feel sorry for me. I don’t have money to go for check-up. I have the sickness where after I urinate, I see blood and sperms in it and my penis has become small. Because I don’t have money, I am not able to seek treatment and I also have some skin disorder at the back of my neck […]. I have had this sickness for almost 6 years since I have been on the streets. We have girls on the streets too and the boys have been engaging in sexual relations with them therefore giving them diseases. So, if you also sleep with these girls, you get affected. Not long ago, I have been developing skin rashes around my private parts. This sickness has affected my relationship with my friends because if I sleep with a girl and another boy also has sex with her, he will be affected by my sickness […]”

(Young man #7, aged 21).

Although some accounts suggested awareness of the causes of (ill) health, more often participants demonstrated a lack of understanding about their health and various symptoms. This was especially the case for skin conditions and genital symptoms. For example, participants frequently described the presence of rashes and skin conditions without an apparent cause. Young girls described symptoms of ‘white’ (candida), yet often attributed this to eating toffees and sweets. The tendency to tie (inappropriately) sickness to alternative causes may help to explain why, at times, participants dismissed the idea of having a health condition. Such beliefs may have been further supported by the absence of health care treatment or hospitalisation, with participants often equating hospital care as evidence of ill health but which they could not access.

“I have the disease called ‘white’; I do not have any other health condition. Medicine was prescribed for my ‘white’ but I don’t have GHc40 to purchase it […]. I am not treating it because I don’t have money to buy the medicine […]. My friends also have the ‘white’ because they have also been eating sweets […]”

(Young woman #1, aged 15).

“Anytime I urinate I see something whitish coming out of my penis, but I don’t know what that is. I have not gone for any medical check-up. I don’t have any medication”

(Young man #4, aged 17).

### 5.2. Health-Enhancing Practices

Participants described some health-seeking and enhancing practices they adopted in order to alleviate the symptoms they experienced. For example, skin conditions and rashes were often ‘treated’ with home remedies and creams given to them by friends—with varying degrees of effectiveness. Other ‘alternative’ health practices and remedies included the use of blood tonics or herbal mixtures or purchasing medicines from street vendors. Such remedies were used as a more affordable alternative to formal health treatment, which was often deemed out of (financial) reach for participants. Herbal remedies were also favoured over Western medicines, which were often described as ‘cures’ for various ailments. Participants’ accounts of alternative medicines reflected their (uncritical) faith in the healing power of herbal remedies and the practices of herbalists—despite showing little knowledge of how or why such treatments made them feel better and stronger. Resorting to self-medication mainly through herbal preparations is an indication of having knowledge of their health condition and perhaps their preparedness to seek treatment, but without the financial means to access health care treatment. Discussions about hospitals and modern-day treatment were thus framed in economic terms and largely out of reach for these young people. Because of this, participants favoured, and accessed, herbal remedies for their symptoms.

“I have not been to the hospital, but I buy blood syrup from one man we call ‘Doctor’. I buy this blood syrup; I have never been to the hospital for check-up […]. I don’t have money to buy medicine”

(Young man #3, aged 16).

“I have not been to the hospital for medical assistance, but when I hear of herbal medicines, I buy ‘times herbal mixture’. We drink them and it is good for our health. We don’t like the foreign medicine because it was these herbal medicines that our elders used to support their health. We are following our elders. When we urinate after drinking the herbal medicine, you see that things are coming out of the body given the colour of the urine. It makes you feel strong”

(Young woman #3, aged 18).

Despite highlighting a lack of understanding about health, most participants did display an awareness of the importance of good hygiene, adequate sleep, diet and exercise—perhaps, in part, reflecting dominant health promotion narratives. Girls, in particular, talked about the importance of personal hygiene, eating fruits, physical exercise and getting adequate sleep to ‘keep healthy’ and be strong—often undertaking such practices with friends.

“I usually sleep to support my health after I engage in any hard work. I also eat a lot of banana to support my health and sometimes go for a walk with my friends”

(Young woman #5, aged 19).

“I buy fruits like apple and banana to eat with my friends. When I get money to buy the fruits to eat it helps me to stay healthy”

(Young woman #1, aged 15).

However, perhaps unsurprisingly, a lack of money meant that their engagement in such practices was often limited. For example, participants described how they could not afford to buy ‘healthy’ food and knowingly ate contaminated food from the streets or were unable to afford the costs of public bathing facilities to wash. For others, the effects of working very long hours in the hot climate without adequate food and water took its toll– highlighting the limits to these young migrants’ opportunities to support their health because of the ongoing and multi-dimensional effects of poverty. Knowledge of (ill) health alone is not enough to stay and be healthy in the absence of the (financial) means to enact health.

### 5.3. Support for Health—The Role of Friends

As evidenced, rarely did participants describe times when they had sought out medical help or hospital treatment for their symptoms, and largely because accessing formal health care was deemed too costly. As discussed, although participants reported having no health issues, on closer examination these apparent contradictions seemed to refer to a lack of access to health care, rather than the absence of illness. Limited access to formal health care meant that participants sought help from friends—who were talked about positively for their contribution to physical and mental health. For example, friends often provided participants with food or supplied medication or other remedies during times of illness. Taking part in activities together such as going to the beach or jogging with friends were described as supporting participants’ health.

“My friends have been helping me to stay healthy. They give me food to eat, when they buy fruits, we eat together. Then we will jog at the shores of the sea together. I don’t go to the hospital for any medical support to stay healthy. I don’t know how the food that my friends give me helps me to stay healthy. It is when I don’t have money and hungry that my friends do buy food for me to eat”

(Young woman #1, aged 15).

Such dependence on friends’ support could, however, be counter-productive in situations where medical intervention is needed. Yet, these friendships were overwhelmingly valued for the positive support they offered for participants’ physical and mental health and their engagement in health-enhancing practices.

Participants often described how friends had positive impacts on their mental health and wellbeing. For example, friends often provided companionship and emotional support during difficult times. Having someone to talk to further signalled the importance of friendships and other social networks—pointing to the importance of enhancing forms of social capital for young migrants to support their health.

“I become happy and feel healthy when I am talking to my friends and when we think together. I tend not to feel healthy when I am alone”

(Young woman #4, aged 18).

Despite these positive reports, some participants highlighted the negative impacts of ‘bad’ friendships on their health. Young men in particular talked about friends smoking, drinking and engaging in drug use—often highlighting a desire to distance themselves from these ‘risky’ health practices and friends. Getting into fights and the experience of theft were also reported as negative peer influences—highlighting the ways ‘friends’ could be both good and bad for health, but also how participants were often dependent on friends to survive. During these discussions, these young men highlighted their worries about being exposed to harmful health practices and behaviours—and thus offered a counter perspective on their friendships and by suggesting a lack of trust.

“For the friends that I have, I don’t trust them. Anytime that they come around they always want to fight and I don’t like that, they also like stealing […]. The friends I have in Accra are all smokers and I don’t like that […]. I don’t like the behaviour of the boys around here, they like begging and fighting. There are a lot of stealing. The way my friends are smoking while I don’t smoke worries me. I wish all of us don’t smoke”

(Young man #6, aged 18).

The complexity of young migrants’ social relationships and friendships was also echoed in young women’s accounts—albeit with a differing focus. In contrast to a focus on health risk behaviours such as smoking, the negative experiences cited by young women suggested difficult personal relationships with boys and abusive partners.

“The boy that I live with maltreats me. The boy has been beating us and my friend will tell me not to mind him and will also be buying medicine for me. The thing that doesn’t make me stay healthy is when the guy maltreats me, he has been beating all of us. Besides if he tells us to wash his clothes for instance and after washing, we move out to go and watch TV, he starts to beat us when we come back saying why did we go out to watch TV. We sleep in one of the kiosks along the beach. He made the kiosk. So, if he beats me and I don’t take medicine I become weak and sometimes I do collapse”

(Young woman #2, aged 17).

“I don’t stay healthy when my boyfriend fights with me. He hits me and one time he scratched my neck with his nails. We usually fight when he does things that I dislike. For instance, he can bring a new girl and tell me that’s his new girlfriend, we then exchange words which finally turns into a fight. With this, if I am working and have my own money, my boyfriend can’t beat me, it is only when I am not working that he can maltreat me”

(Young woman #5, aged 19).

These detailed accounts suggest a complex inter-dependency within their personal relationships and highlight the gendered inequalities that affect these young migrants. Girls appeared especially vulnerable, as they were often dependent on boys and partners for food and shelter, all the while wishing to distance themselves from abusive relationships. Boys too were subject to dominant gender norms as they described the need to assert themselves physically, engage in fights and steal in order to protect their livelihoods and ultimately ‘survive’. Such accounts underscore the persistent and multi-dimensional and gendered impacts of poverty, including how the broader social determinants of health shape young migrants’ everyday lives and the (lack of) realistic opportunities to support their health. Inevitably, participants called for more help with finding meaningful employment or support with returning to education in order to lift them out of poverty and offer a pathway to a better life and positive (future) health. Unfortunately, this desire to go back to school or find a good job may never be realised and given the complex vulnerabilities these young people experience.

“What I need is someone to help me learn catering; we are all willing to learn it if someone should help us. If I get money, I will use it to buy and sell sausage and then use the profit to buy the items that I need to learn the catering. I will also need someone to support me buy the items to help me learn catering”

(Young woman #5, aged 19).

## 6. Discussion

Research exploring health amongst African migrants is limited, particularly with younger populations [39]. Even less work has focused on possibilities for health promotion with young migrant groups—particularly in Africa, including detailed exploration of the ways young migrants engage with (or not) health-enhancing practices. Our research offers crucial new insights into the health practices and experiences of young migrants who have migrated internally to the capital city in Ghana. Specifically, our findings demonstrate how the multidimensional and gendered impacts of poverty and broader social determinants of health shape young migrants’ opportunities to support their health. Indeed, the experiences of disadvantage were found to negatively influence access to basic health needs including a safe space to sleep, adequate food and water, and access to formal health care. Such findings build on the scant evidence base that examines the health of young migrants in Ghana [6] and underscores the critical importance of migration to health—including how broader determinants of health intersect to impact migrants’ health experiences [8,40]. Increased exposure to health risks and ‘risky’ health practices was evident as young people navigated precarious living and working conditions that directly affected their health—often with little choice but to continue, all the while knowing the harms to health.

Health promotion underscores the importance of inter-sectoral collaboration and policy responses that tackle multiple determinants of health, including poverty reduction strategies as well as enhancing access to health-enhancing practices and environments and formal health care [21,22]. As suggested by participants, creating meaningful employment opportunities to support young migrants’ livelihoods may be a necessary step towards positive health. Such opportunities may enhance the socio-economic circumstances of young migrants and enable them access to secure shelter and food. Further, our results suggest the relevance of expanding training and education programmes that enhance opportunities for young migrants to learn new skills and trades that advance their livelihoods as a pathway to positive health outcomes and advancing health literacy.

In this study, lack of access to health care was especially pronounced with few participants sharing examples of times when they had sought out treatment or diagnosis from a qualified health professional. Ghana has had a national health insurance policy and scheme since 2003 for all persons in the country. Yet, evidence of poor uptake of formal health care has been described in other research that demonstrates the difficulties young people have registering for the National Insurance Health Scheme in Ghana—and because parents are required to register their children [6], but also because of understandings of health and illness. For example, there is a widespread tendency for people in Ghana to assume health insurance is needed only during times of illness [41] and those uninsured do not access health care unless they are seriously ill [42]. Participants’ descriptions of ‘having no health complaints’ may thus, explain low uptake of the NHIS—despite contradictory reports of a range of symptoms. Such contradictions are likely to impact on the relative successes of health promotion approaches and their perceived relevance to young migrants—particularly if they do not see themselves as being ill or requiring formal treatment.

Participants’ apparent reluctance to register for formal health care may further explain why they more often self-diagnosed and self-medicated common symptoms and ailments—often with limited understanding of the aetiology of symptoms or effectiveness of medications given to them by friends or herbalists. In some ways, self-medicating against illnesses and other techniques can be seen as one way that participants sought to take control over and promote their health within the constraints of their living and working conditions [6]. Whilst examples of this kind may suggest evidence of young people’s health agency, such practices also raised concerns about the safety and efficacy of self-medication of undiagnosed conditions. This seemed especially the case for genital-urinary symptoms that pointed to possible sexually transmitted infections. Enhancing access to health care, including screening, is thus critical to support these young people’s self-management of health and illness; yet, as evidence here suggests, this needs to be firmly coupled with policy responses that address broader economic influences on migrants’ health—highlighting the importance of ensuring ‘Health For All across’ different sectors [43], not just health care.

Despite highlighting negative influences on their health, participants did purposely engage in activities to support their health, such as exercise and eating fruits and demonstrated some understanding of nutrition and personal hygiene to their health. In part, these accounts may reflect uptake of dominant health discourses, rather than their actual health practices [26]. Yet, at other times, participants displayed a lack of functional health literacy—defined as the ability to access, understand and importantly apply health information [44]—which again may explain why formal health care was seldom accessed. Indeed, participants were often unable to articulate the aetiology of symptoms or how particular medications might work– both of which are likely to be exacerbated by poor access to health care and advice from health professionals. Whilst approaches to enhancing health literacy amongst young migrants in Ghana would be a useful step towards improving health knowledge and outcomes, such approaches would not be enough in themselves to circumvent the persistent effects of poverty and the lack of access to key assets and resources that support health, including meaningful employment and safe places to live and work.

Support for health was overwhelmingly described by reference to the role and importance of friends, who were often relied upon to provide food, shelter and medications during times of hardship [6]. Such findings highlight how health is embedded in social relationships and the importance of these relationships to supporting both the physical and mental wellbeing of vulnerable young people [5]. Harnessing ways to deepen social relationship and build social capital for young migrants may be a useful starting point for health promotion with young migrants [2]. Yet, as our findings illustrate, friendships held negative impacts too, with both young men and women describing difficult social relationships that hindered their health. The gendered variations in friendships highlighted the particular vulnerabilities experienced by girls as they described examples of abusive and exploitative relationships with boys. Such vulnerabilities have been reported elsewhere and are often reflected in girls’ increased risks of STIs, pregnancy and abuse [6]. However, our work points to the complex inter-dependency of these relationships, whereby economic factors such as poverty and limited employment options exposes these girls to a relationship of dependency as they often relied on boys for food and shelter to survive. Such gender disparities reflect the urgent work needed to address Goal 5 of the SDGs and secure pathways to gender equality. This includes supporting young men to find meaningful employment and livelihoods to mitigate the impacts of poverty and reduce their exposure to harmful practices, including violence against girls. Ultimately, joined up action across a number of sectors (health, education, economic, housing) is urgently needed to create sustainable environments that enhance, rather than hinder, opportunities for positive health.

## 7. Conclusions

Migration of young people from other countries within the West African region to Ghana and within Ghana is a common phenomenon and is largely driven by economic reasons. Many migrants (both internal and international) live in vulnerable socio-economic situations with significant impacts on their health and health-seeking practices, as our findings illustrate. Key lessons learnt present valuable bases for health promotion in the context of the SDGs and their indivisible nature. Action is needed across all Goals (and especially 1–4, 8 and 11) to support young migrants’ health and lives. Supporting positive livelihoods as part of tackling the social determinants of health is needed to mitigate the impacts of poverty and inequalities on young migrants’ health practices and outcomes. Crucially, the perspectives and health experiences of young migrants who live and work in vulnerable contexts and circumstances must be included in policy discourses and debates that work towards achieving the SDGs and secure positive pathways to health for all.

## Data Availability

Not applicable.
